# Aluminum Stress Induces Irreversible Proteomic Changes in the Roots of the Sensitive but Not the Tolerant Genotype of Triticale Seedlings

**DOI:** 10.3390/plants11020165

**Published:** 2022-01-08

**Authors:** Agnieszka Niedziela, Lucyna Domżalska, Wioletta M. Dynkowska, Markéta Pernisová, Krystyna Rybka

**Affiliations:** 1 Department of Biochemistry and Biotechnology, Plant Breeding and Acclimatization Institute-National Research Institute in Radzików, 05-870 Blonie, Poland; w.dynkowska@ihar.edu.pl; 2 Center for Biological Diversity Conservation in Powsin, Polish Academy of Sciences Botanical Garden, Prawdziwka 2, 02-973 Warsaw, Poland; lucyna.domzalska@gmail.com; 3 Plant Sciences Core Facility, Mendel Centre for Plant Genomics and Proteomics, Central European Institute of Technology (CEITEC), Masaryk University, Kamenice 5, 62500 Brno, Czech Republic; marketa.pernisova@ceitec.muni.cz; 4 Laboratory of Functional Genomics and Proteomics, National Centre for Biomolecular Research, Faculty of Science, Masaryk University, Kamenice 5, 62500 Brno, Czech Republic

**Keywords:** acidic soils, abiotic stress tolerance, proteomic studies, two dimensional electrophoresis (2-DE), × *Triticosecale* Wittmack

## Abstract

Triticale is a wheat–rye hybrid with a higher abiotic stress tolerance than wheat and is better adapted for cultivation in light-type soils, where aluminum ions are present as Al-complexes that are harmful to plants. The roots are the first plant organs to contact these ions and the inhibition of root growth is one of the first plant reactions. The proteomes of the root apices in Al-tolerant and -sensitive plants were investigated to compare their regeneration effects following stress. The materials used in this study consisted of seedlings of three triticale lines differing in Al^3+^ tolerance, first subjected to aluminum ion stress and then recovered. Two-dimensional electrophoresis (2-DE) was used for seedling root protein separation followed by differential spot analysis using liquid chromatography coupled to tandem mass spectrometry (LC-MS-MS/MS). The plants’ tolerance to the stress was evaluated based on biometric screening of seedling root regrowth upon regeneration. Our results suggest that the Al-tolerant genotype can recover, without differentiation of proteome profiles, after stress relief, contrary to Al-sensitive genotypes that maintain the proteome modifications caused by unfavorable environments.

## 1. Introduction

Aluminum is the third most abundant element on earth, after oxygen and silicon. Its toxic effect in plants results from the physicochemical properties of common aluminum minerals, presented in the lithosphere as, for example: gibbsite and bauxite (hydroxylated Al-ions), kaolinite, or muscovite (hydrated complexes of aluminum and potassium). All minerals containing aluminum are insoluble at a neutral pH (6.5–7.0); hence, aluminum ions in such soils are biologically passive, non-available and thus non-harmful to plants. In acidic (pH 5.0–6.0) or very acidic (pH 4.0–5.5) soils, aluminum containing minerals can become soluble, releasing hydroxyl complexes of Al-ions in trivalent cationic forms, which are complexed in humus soils but picked up by plant roots from acidic sandy soils [1]. Since acidic soils constitute 30–40% of the world’s arable land, with a constantly growing share due to anthropogenic impact, crop plants’ aluminum tolerance is one of the features that affect higher/stable yielding in changing environments [2]. 

Tolerance to Al-ions relies on the inhibition of Al uptake by the roots (external tolerance) and/or on the inhibition of transport to the aerial parts (internal tolerance). In fact, most of the tolerant crop plants are Al-exuders, avoiding the stress by prevention of ion intake into the symplast [3]. Their basic mechanisms are citrate, malate, and/or oxalate secretion into the rhizosphere for chelating Al-ions into non-absorbable complexes as well as a pH increase in the rhizosphere as an effect of plasma membrane H^+^-ATPase activity, higher in the roots of tolerant plants [4]. The base of the internal tolerance is Al-cation binding by negatively-charged carboxyl groups of the plant cell wall pectins in the root apex. Pectin content and the degree of methylation differentiate the Al tolerance response [5]. Another mechanism for Al tolerance is ion chelation in the cytosol and relocation to leaf vacuoles. Al-ions are present mostly in hydroxylated forms in the apoplast, whereas in symplast, they form complexes with sulfate, phosphate, and organic ligands [2]. When the tolerance mechanisms fail, the Al-ions gradually move inside the cells, misbalancing and eventually blocking ion channels, alternating lipid fluidity, and inducing changes in the cytoskeleton structure by binding to G-proteins and their substrates as well as to ATP-ases and nucleotide polyphosphate groups. Finally, the disruption of DNA synthesis and cell division in the root apex and the lateral roots is accompanied by increased rigidity of the cell walls and DNA double helix, which leads to rapid inhibition (within an hour) of root growth, even with micromolar Al^3+^ concentrations [2,6]. The biochemical consequences of aluminum intake are an increase in reactive oxygen species, increased fatty acid peroxidation, and inhibition of proton adenosine triphosphatase H^+^-ATPases. The displacement of Ca^2+^ in cell membranes contributes to Al^3+^ accumulation in the apoplast, stimulates callose synthesis, and finally inhibits intercellular transport. The decreasing concentration of Ca^2+^ in the cytosol alters the pH balance, which in turn interferes with sugar phosphorylases and the deposition of cell wall polysaccharides [2,6,7]. 

The genetic bases of Al tolerance concern ion chelation and ion transport. The gene families responsible for Al-ion exudation are ALMT (aluminum-activated malate transporter), responsible for malate, and MATE (multidrug and toxin efflux) for citrate exudation [8,9,10]. The transcriptional expression of ALMT and MATE is controlled by a master zinc-finger transcription factor, STOP1 (sensitive to proton rhizotoxicity 1) [9,11]. Internal Al tolerance is less well characterized. The NRAT1 (Nramp aluminum transporter 1) transporter has been identified as a putative Al transporter involved in rice’s internal resistance mechanism, which lowers Al-ion concentrations in the root cell wall, transporting the ions inside the root cells for sequestration in vacuoles [12]. Those genes are conserved in numerous plant crop genomes [13]. The STOP1 transcription factor, in addition to several phytohormones, hydrogen peroxide, and other reactive oxygen species (ROS), take part in the upregulation of genes of the ALMT family or root growth inhibition. In addition, ROS detoxifying enzymes are activated to respond to aluminum ion stress, activating the gene network towards the induction of aluminum tolerance in plant tissues [13].

Hexaploid triticale, a rye–wheat hybrid species of wheat (AABB genomes) and rye (RR genome), is characterized by an intermediate tolerance between wheat considered as an Al-sensitive and rye as an Al-tolerant parent. The beneficial influence of the rye genome was confirmed by Quantitative Trait Loci (QTL) localization on the 7R chromosome, explaining up to 36% of the phenotypic variance, including the malate transporter gene [14]. The other loci were found on chromosomes 3R [15,16], 4R, and 6R [15], without a recognized function in the triticale genome. Triticale’s tolerance to Al-ions is less than that of rye, suggesting a suppressive effect of the wheat genome on the expression of rye genes.

In the present work, two-dimensional electrophoresis (2-DE) with immobilized pH gradients (IPGs), combined with protein identification by mass spectrometry (MS), was used to detect changes in the proteomes of triticale root tips after Al stress removal. This method has been successfully used to identify proteins involved in various stress responses in plants [17,18]. Al-responsive proteins identified in both monocots [19,20,21,22] and dicots [23] were functionally associated with cell division and structure, carbohydrate metabolism, antioxidant system, amino acid metabolism, protein degradation, signal transduction, and transporters [21,24,25]. The upregulation of the enzymes involved in cysteine and methionine metabolism, such as cysteine synthase, S-adenosylmethionine synthase, and O-methyltransferase, is a common response despite Al-ion tolerance [21,22,23,25,26]. These enzymes are required to maintain methyl cycling and glutathione metabolism, which are important mechanisms that lead Al detoxification [27]. The recent proteomic studies of soybean plasma membrane changes in response to Al-ions revealed about fifty different membrane traffic and transporter proteins [28]. 

To our knowledge, there are no reports concerning protein identification in triticale roots in the context of aluminum tolerance; however, the triticale root proteome response to drought was studied by Grębosz et al. [29]. For our comparative proteomic studies, we used the seedling root tips of triticale plants differing in Al tolerance subjected to recovery after the stress treatment. 

## 2. Results

### 2.1. Biometric and Biochemical Evaluation of Tested Materials

**Figure 1:**
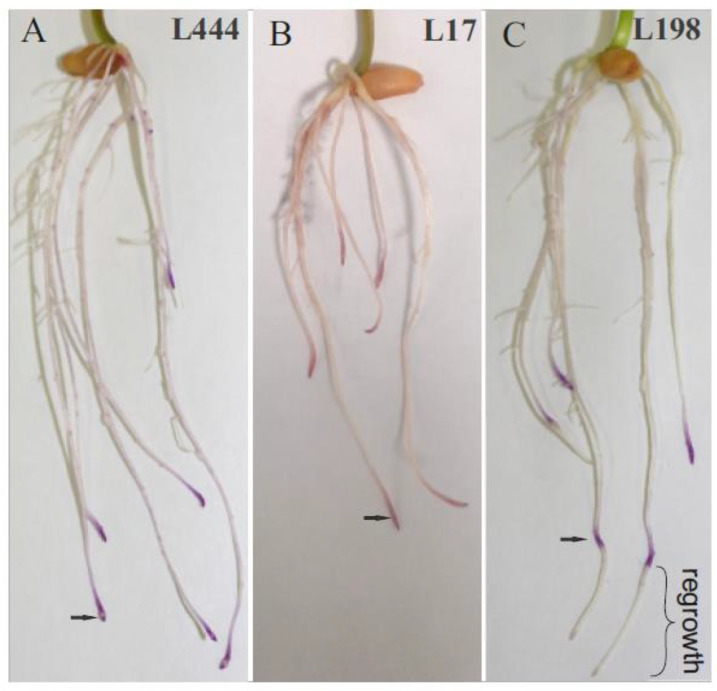
Damaged regions of triticale seedling roots (denoted by arrows) stained with Eriochrome cyanine R after Al-ion treatment prior to recovery. After 48 h recovery, the purple root tips and no rooth regrowth were visible in case of the Al-sensitive lines (L444 and L17 (**A**,**B**), respectively), whereas the dark purple bands on regrown roots were detected in the case of the Al-tolerant line (L198 (**C**)).

The materials used in the studies were selected based on a biometric screening of 232 triticale lines in earlier published experiments [15] (Figure S1). One Al-tolerant line (L198, spring form) and two Al-sensitive lines (L17, spring form and L444, winter form) were chosen for the present studies. Root growth in response to 24 h of Al treatment was inhibited and in sensitive lines the root regrowth remained suppressed 48 h after the stress release, contrary to the roots of tolerant line, which resumed root growth after the stress release (Figure 1). Eriochrome cyanine R dye penetrates damaged or partially damaged root tips. The root regrowth ranged from 0.3 to 2.5 cm for individual seedlings of the tolerant line (Table 1). Additionally, the root apex redox potential of the seedlings released from Al stress was assessed in comparison with the control seedlings as a measure of the dynamics of the response to the Al stress removal [30]. Antioxidant capacity was determined using 2,2-diphenyl-1-picrylhydrazyl anion radical (DPPH*^−^), and the cation radical 2,2′-azino-bis(3-ethyl benzothiazoline-6-sulphonic acid (ABTS*^+^) [31]. According to one-way analysis of variance (ANOVA), no significant differences (with *p* ≤ 0.05) were detected between the control and stress treated seedlings in the case of reactions with DPPH*^−^ anion radical, despite a 20% difference in the L444 line between the control and Al-treated samples (Table 1). In the case of reactions with ABTS*^+^, 13% higher activity, with statistical importance at *p* ≤ 0.05, was detected after the stress release in the case of the L444 line, and 10% (with no statistical importance) in the case of the L17 line. There were no differences in redox potential between the control and treated roots of the tolerant line L198 (Table 1). 

### 2.2. Two-Dimensional Electrophoresis (2-DE)

The analysis of 2-DE gels revealed approximately 590 spots in each experimental and biological replication of both the control samples and those stress released after the Al-treatment (Table 2). Ninety-five percent of the protein spots were matched and quantified. Isoelectrophocusing using IPG strips of pH 3–10 revealed the protein spots at pI values in the range of 4.0–8.5 and protein masses between 6.5 and 95 kDa (Figure S2A–F, Table 3). In the tolerant line (L198), the proteomes of stress-subjected and control roots were not differentiated according to the established criteria (*p* ≤ 0.01 and difference in spot intensity ≥ two-fold). When the criterion of probability was weakened from 99% to 95%, only four differential protein spots were found (three spots as downregulated and one spot as upregulated). On the other hand, in the root tip proteomes of the Al-sensitive triticale lines, a higher number of differentiated protein spots were found. Regardless of the protein spot intensity, with a *p* ≤ 0.01 probability criterion, in total seventy-one differential protein spots were found in the L17 proteome (23 upregulated, 21 downregulated, 15 induced, and 12 silenced) and forty-three in the L444 proteome (23 upregulated, eight downregulated, three induced, and nine silenced). When the criterion of two-fold difference in spot intensity was added, 14 upregulated and eight downregulated spots were found in the L17 proteome. In L444, upon a double criterion (probability and spot intensity), 18 spots of upregulated proteins, exclusively, were found. For induced or silenced protein spots, a second criterion of spot intensity ≥ 0.2 was decided. In the proteome of L17, nine spots had a relative signal intensity > 0.2 among induced proteins, and three among those silenced, whereas in the proteome of L444 root tips, three induced and nine silenced protein spots were found (Table 2, Figure S3).

The highest, nearly 8-fold, difference in upregulated protein spot intensity was detected for spot #6, and a 5.2-fold decline for #24 were detected in the L17 proteome (Table 3, Figure S2B). In the L444 proteome, a spot identical to spot #6 was induced de novo in response to Al stress with a relative intensity of 0.26, and was numbered as spot #31 (Table 4, Figure S2D). Furthermore, seven of the differential proteins (numbered: 2, 4, 9, 12, 16, 17, and 18 in the proteome of L17 and 3, 8, 13, 14, 15, 19, and 21 in the proteome of L444) showed a more than two-fold increase in signal intensity in the L17 proteome and about a two-fold increase in the L444 proteome (Table 3, Figure S2B,D). Ten spots revealed significant differentiation (with *p* ≤ 0.01) for one of the two lines. Out of the eight protein spots of L17 that were downregulated with two-fold or higher intensities, none differed in this intensity in L444 (Table 3). This number of spots was counted according to the acute cut-off double criterion (probability *p* < 0.01 and intensity difference > 2); however, with weakened criteria and a single cut-off (probability *p* < 0.05), more than 80% of the protein spots were common for L17 and L444 lines. Since the spot identification was performed according to a cut-off by strong, double criteria, the weaker criteria data are not shown in detail (Table 3 and Table 4).

### 2.3. Identification of Differential Proteins

The identification of differential proteins was carried out using liquid chromatography coupled to tandem mass spectrometry LC-MS-MS/MS system for 25 selected spots, with at least a two-fold change in intensity for at least one of the Al-sensitive lines (Table 3). Differential proteins from the spots marked as identical by the gel analysis software (Image Master 2D Platinum 7.0) were analyzed, except three upregulated protein spots (#2, #16, and #17) that represented both sensitive lines L17 and L444. The spots were extracted from the 2-DE gels followed by the separation of L17 and L444 root tip proteomes. Moreover, six induced and five silenced protein spots, with a signal intensity ≥ 0.2, were identified. In total, out of 36 protein spots, thirty-two represented 23 differentially expressed proteins, whereas three were unassigned (#24 and #25 from L17, and #36 from L444) (Table 3 and Table 4).

**Figure 2:**
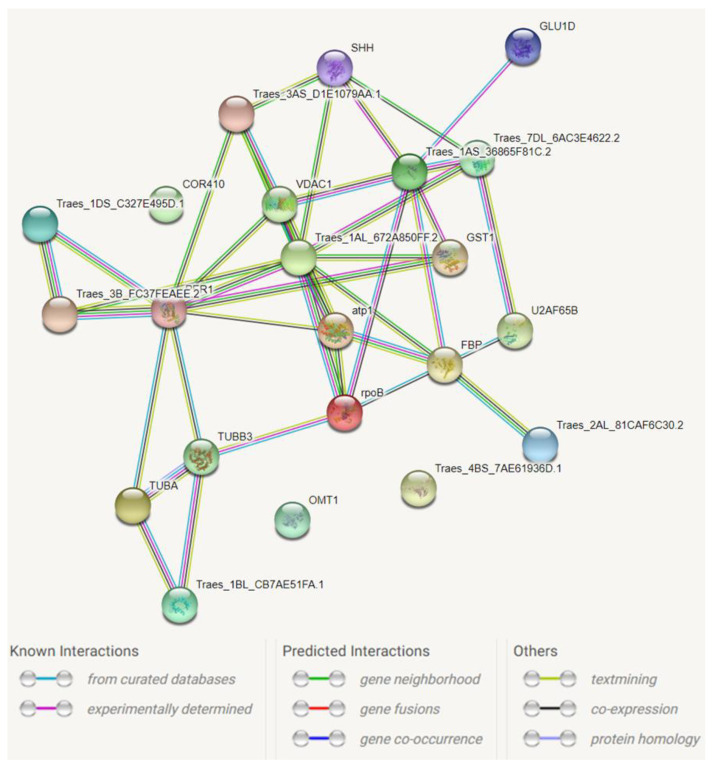
Computational prediction of the functional network between differential proteins. The proteins used for analysis are presented in Table 3 and Table 4. The number of connecting lines is in proportion to the amount of information about the protein interactions available. The line color indicates the type of interaction evidence. The explanation of symbols used in STRING database annotations are as follows: Traes_4BS_7AE61936D.1—oxalate oxidase; GST1—glutathione S-transferase; atp1— ATP synthase subunit alpha, mitochondrial; PER1—1-Cys peroxiredoxin; Traes_3B_FC37FEAEE.2—protein disulfide-isomerase; U2AF65B—splicing factor U2af large subunit B; Traes_1DS_C327E495D.1—serpin-Z1C; Traes_7DL_6AC3E4622.2—eukaryotic initiation factor 4A; Traes_1BL_CB7AE51FA.1—calmodulin; Traes_1AS_36865F81C.2—ubiquitin; SHH—adenosylhomocysteinase; Traes_1AL_672A850FF.2—phosphoglycerate kinase, cytosolic; GLUD1—DIMBOA 1b, chloroplastic; TUBB3—tubulin beta-3 chain; TUBA—tubulin alpha chain; Traes_3AS_D1E1079AA1—S-adenosylmethionine synthase; VDAC1—mitochondrial outer membrane porin; FBP—fructose-1,6-bisphosphatase; COR410—dehydrin COR410; rpoB—DNA-directed RNA polymerase subunit beta; OMT1—flavone O-methyltransferase 1; Traes_2AL81CAF6C30.2—phosphomannomutase.

The identified proteins represented ten protein functional groups involved in cell division, protein folding, protein synthesis, stress-related response, metabolic pathways, lignin synthesis, transcription control, protease inhibition, protein degradation, and transport (Table 3 and Table 4). The highest upregulation was detected for the protein folding (protein disulfide-isomerase), stress-related response (glutathione S-transferase, oxalate oxidase, 1-Cys peroxiredoxin PER1), and metabolic pathways (flavone O-methyltransferase 1). On the contrary, the downregulated proteins belonged to the cell division (tubulin) and metabolic pathways associated with amino acid metabolism and methylation control (adenosylhomocysteinase) as well as ascorbic acid biosynthesis (phosphomannomutase). The Protein-Protein Interaction Networks analysis (using STRING database) [32] revealed a functional network containing 19 nodes (flavone O-methyltransferase 1, oxalate oxidase, and serpin-Z1C were not connected) with 39 edges (vs. 34 expected) (Figure 2). We discovered two major proteins (1-Cys peroxiredoxin and phosphoglycerate kinase) with nine interactions in the network. Moreover, seven interactions were detected for ubiquitin.

## 3. Discussion

### 3.1. Evaluation of the Al Stress Response of Tolerant (L198) and Sensitive (L17, L444) Triticale Lines

The earliest symptoms of Al toxicity concern the meristematic zone in the root apex [6,7,33], which results in the inhibition of root growth and, finally, in declined crop yield. Such root damages, confirmed by microscopic studies, have been described for many plant species [34,35]. Our experiment, performed in the frame of Al tolerance biometric phenotyping [36] developed for breeding selection purposes, also showed the inhibition of seedling root regrowth (Figure 1). This method enables one to distinguish the Al-tolerant genotypes, without completely damaging the root meristems [37]. The test developed by Aniol [37] is broadly used in the breeding selection of cereal crops. Depending on the plant species, different concentrations of Al-ions are used [36]. For triticale, which is less tolerant than rye but much more tolerant than wheat, a 16 ppm ion concentration is common and allows for clear differentiation between tolerant, intermediate, and sensitive lines, among which the tolerant forms are in minority [14,36,38]. Recent studies by Szewińska et al. [35], performed in line with the biometric phenotyping method, revealed that after the Al stress release, the epidermal cells of root tips in tolerant rye and triticale seedlings are replaced by new cells and the root growth is maintained [35], and this process is independent on organic acid exudation [39]. In our studies, the roots of Al-tolerant L198 triticale line regrew, and the proteomic data showed identity between the control and root tips recovered after the stress release. In contrast, the root tips of susceptible lines were permanently damaged and roots did not regrow, along with evidenced alterations between the control and stress-treated proteomes, which is in line with the literature data [24]. 

Additionally, the detected differences in the redox balance in root tips showed complete recovery in the case of the tolerant line after the stress release, and differentiation between the control and stress treated seedlings in the case of the susceptible lines [30]; however, these differences in the present experiment carried out according to the biometric test protocol were found only in the case of line L444 root extract reactions with the ABTS*^+^ cation radical (Table 1). The proteomic data were analyzed with double, strong cut-off criterion (probability *p* ≤ 0.01 and difference in intensity ≥ 2), which influenced the small number of identities between the proteomes of L17 and L444. When the criterion was weakened and the intensity ratio was neglected, more than 80% of proteome patterns were common for L17 and L 444 lines (Table 2). 

### 3.2. Annotation of Protein Spots

The flavone O-methyltransferase 1 (OMT1), with homology to the *Triticum aestivum* enzyme, was the strongest upregulated protein found in triticale root tips of the L17 sensitive line, whereas in L444 it was synthesized de novo. This enzyme catalyzes the sequential O-methylation of tricetin to mono-, di-, or trimethylated derivatives, with tricin, a dimethyl derivative, as a component found in monocotyledonous lignins [40]. Cell wall lignification along with hemicellulose deposition is an important and well-documented mechanism of plant tissue protection against harmful Al-ions, is positively correlated with the inhibition of root elongation, is more strongly expressed in sensitive genotypes [41], and was also detected in our experiment. *Ta*OMT1 was initially considered as a putative caffeic acid O-methyltransferase [42] involved in lignin biosynthesis; however, Zhou et al. [43] documented its low activity in methylation of lignin precursors such as caffeic and 5-hydroxyferulic acids. The next intensity difference was assigned to oxalate oxidase (OXO). Despite the fact that oxalate synthesis and degradation in plant cell walls is not clearly understood [44], it is specified as the enzyme oxidizing the oxalate to CO_2_ and H_2_O_2_. It was reported that an Al-induced increase in OXO was correlated with Al uptake, growth inhibition, damage of the plasma membrane, and disruption of membrane permeability in barley seedling roots [45]. The increased activity of OXO has been observed in the roots of barley [45] and wheat [21]. The increased concentration of H_2_O_2_ disturbs cell redox homeostasis, leading to the activation of stress response pathways on the one hand and apoptosis on the other. Delisle et al. [46] concluded that a high level of OXO expression may support trapping the Al-ions in the root cells rather than induction of H_2_O_2_-dependent cell death, which was observed in wheat epidermal cells after only 8 h exposure to Al. The other antioxidant enzymes, glutathione-S-transferase (GSH) and 1-Cys peroxiredoxin (PER1), were also found. GSH is known as a universal antioxidant and detoxifier, induced in response to various stresses [47]. It is one of the most common enzymes identified in protein and transcript analyses of different plant species exposed to Al stress, with increased activity in both Al-tolerant and -sensitive genotypes of soybean [23], flax [48], maize [49], *Arabidopsis* [27], and pea roots [50]. However, an unexpected suppression of GSH protein was also observed in tomato (−1.56-fold) and wheat (−2.5-fold) [21,43]. 

The identified proteins S-adenosylmethionine synthase and adenosylhomocysteinase are enzymes of methyl cycling. S-adenosylmethionine synthase (SAMS) catalyzes the formation of S-adenosylmethionine (SAM) from methionine and ATP. Adenosylhomocysteinase may play a key role in the control of methylations via regulation of the intracellular concentration of adenosylhomocysteine, an inhibitor of SAM-dependent methyl transferase reactions. In earlier proteomic studies, a dynamic induction of SAMS in Al-treated roots of wheat [21], tomato [26], and rice [25] was found. The same analysis showed downregulation of adenosylhomocysteinase in wheat [21]. It was proposed [51] that stimulation of SAM synthesis could be involved in the alteration of the cell wall and polymer structures in roots and/or ethylene-mediated inhibition of root growth. S-adenosylmethionine (SAM) may also serve as an important methyl donor for O-methyltrasferases (OMT), involved in lignin synthesis [52]. Due to the fact, that the synthesis of DIMBOA-Glc requires O-methylation catalyzed by O-methyltransferases with the presence of SAM as methyl donor, we speculate that methyl cycling also plays an important role in DIMBOA synthesis in triticale plants exposed to Al stress. The increase or de novo synthesis of DIMBOA (2,4- dihydroxy-7- methoxy-1,4- benzoxazin-3-one) glucosidases, GLU1b and GLU1c, in protein extracts from Al-sensitive root tips suggests that the hydrolysis of terminal, non-reducing beta-D-glucosyl residues releases the DIMBOA benzoxazinoid, a key defense compound, along with the DIBOA (2,4-dihydroxy-1,4-benzoxazin-3-one), present in major agricultural crops, such as maize and wheat, and biologically active in both the above-ground and underground parts of plants. Poschenrieder et al. [53] documented its role in maize root tip protection by chelating Al-ions in the rhizosphere, and Neal et al. [54] found their attractive function for *Pseudomonas putida* in the maize. The inhibition of root growth entails enhanced cell wall rigidity [7] and changes in the organization of cortical microtubules [55]. A significant decrease in α- and β-tubulins, the main components of microtubules, was observed in proteomes of both sensitive lines. Similar results were obtained for Al-sensitive maize [56] and rice [20] under Al stress. Interestingly, different subunits of tubulin were differentially expressed and changed dynamically in the Al-sensitive soybean [23].

A significant induction of several proteins involved in protein synthesis and degradation was observed as well. Among them, DNA-directed RNA polymerase subunit beta, which catalyzes the transcription of DNA into RNA, as well as splicing factor U2af large subunit B, necessary for the splicing of pre-mRNA, were upregulated. Moreover, we found a high induction of the eukaryotic initiation factor 4A (eIF4A), an ATP-dependent RNA helicase that is a subunit of the eukaryotic translation initiation factor 4F (eIF4F) complex involved in cap recognition, required for mRNA binding to ribosomes [57]. As aluminum stress affects the cellular gene expression machinery, it is evident that molecules involved in nucleic acid processing, including helicases, are likely to be affected in root tips [58]. The two eIF4As/helicases from pea have been shown to play a role in abiotic stress tolerance, especially for salinity and cold stress- [59,60,61]. The expression of *Pennisetum glaucum* eukaryotic translational initiation factor 4A exhibited superior growth performance and higher chlorophyll retention under simulated drought and salinity stresses compared to the control plants. Abiotic stress usually leads to protein unfolding, misfolding, and aggregation [62]. Protein disulfide isomerase-like proteins (PDIs) catalyze protein disulfide bonds, inhibit aggregation of misfolded proteins, and function in isomerization during protein folding in the endoplasmic reticulum and responses during abiotic stresses [63]. In triticale plants affected by aluminum, PDIs were found to be upregulated in both susceptible lines. PDIs from *Brachypodium distachyon* L., *Brassica rapa* ssp. *pekinensis*, and *Arabidopsis thaliana* were upregulated under abiotic stresses, such as drought or salt, as well as under the influence of abscisic acid (ABA), and hydrogen peroxide (H_2_O_2_) an reactive oxygen species, suggesting their involvement in multiple stress responses [62,64,65]. The activation of ubiquitin enzymes illustrates the proteolytic activity in response to Al stress. Ubiquitination plays a critical role in protein inactivation, the degradation of damaged proteins, and the regulation of several mechanisms related to abiotic stress responses [66]. The enzymes of glycolysis and the tricarboxylic acid TCA cycle were activated as well, showing the influence of Al-ions on these main biochemical pathways. The phosphoglycerate kinase, which catalyzes the ADP-dependent dephosphorylation of 1,3-bisphospho glycerate to 3-bispsphoglycerete in glycolysis, was activated. We also observed upregulation of fructose-1,6-bisphosphatase, a key metabolic enzyme that catalyzes the reversible aldol cleavage of fructose-1,6-bisphosphate into glyceraldehyde-3-phosphate, either in glycolysis or gluconeogenesis and in the Calvin–Benson cycle [67]. Stimulating glycolysis in Al-treated plants may accelerate pyruvate and acetyl CoA production for organic acid synthesis, such as citrate or malate, which serve as Al chelators in the tolerant genotypes [7]. Moreover, acetyl-CoA may be used for the synthesis of malonyl-CoA, an essential substrate of fatty-acid synthesis [68]. The regulation of lipid membrane composition and modification of membrane fluidity by changes in unsaturated fatty acid levels is an efficient barrier that prevents metals from entering to the symplasm [69]. The mechanisms of Al tolerance based on increasing the plasma membrane (PM) permeability by binding Al to negative sites on the PM surface of root cells have been well documented for numerous plant species [69,70,71,72]. A similar response of sensitive plants in the regeneration phase may suggest that plants still attempt to eliminate aluminum accumulated in root tips. 

The other proteins, such as mitochondrial ATP synthase, mitochondrial outer membrane porin, calmodulin, and dehydrin COR410 were upregulated in susceptible triticale lines at 48h after Al treatment, which suggest their important role in the response to Al toxicity. Mitochondrial ATP synthase subunit alpha produces the energy storage molecule adenosine triphosphate (ATP), which is suggested to provide energy for active Al efflux and detoxification [22]. Mitochondrial outer membrane porin is responsible for forming a channel through the cell membrane that allows the passage of small molecules. This protein was upregulated in Al-susceptible triticale lines. The abundance change of these mitochondrion transport-related proteins under Al stress indicates that the ion/metabolite exchange between the mitochondria and cytosol was modulated in the roots to cope with the stress. It was also observed that Al induces calmodulin synthesis, a major sensory molecule that decodes Ca^2+^ signals in the presence of different biotic and abiotic stresses [73]. Dehydrins (DHNs) play an important protective role in plant cells during dehydration [74]; however, those containing relatively large amounts of reactive residues on their surface exhibit also reactive oxygen species (ROS) scavenging and metal ion binding properties. However, the role of dehydrin in Al stress has not been explained so far, though its documented properties may suggest a positive correlation with Al tolerance.

Our results indicate that seedlings of Al-tolerant genotypes can recover after 16 ppm Al3+ stress relief without differentiation of proteome profiles (according to criteria: *p* ≤ 0.01 and difference in spot intensity ≥ two-fold), contrary to seedlings of Al-sensitive genotypes that maintain the proteome modifications caused by unfavorable environments. Enzymes involved in cell wall lignification were highly induced whereas proteins involved in cell division were strongly downregulated. 

## 4. Materials and Methods

### 4.1. Plant Materials

The experiments were performed using triticale inbred lines differing in aluminum (Al^3+^) stress tolerance: one Al-tolerant line, L198 (MAH3405 (Milewo) × Matejko), spring form, and two sensitive lines L17 (Gabo × 6944/97), spring form and L444 (MAH3198 × CHD2807/98-7-1), winter form. Seeds were obtained from Plant Breeding Strzelce Ltd., Experimental Station Małyszyn (Poland). The lines were highly homozygotic (F10 generation) and screened for Al tolerance annually in line with our previous and present projects [15,75].

The research was carried out using the common Al tolerance detection method developed by Anioł [37]. Seeds sterilized and germinated for one day to form a 3 mm sprout were sown on polyethylene nets floated in a tray filled with a base medium of 2.0 CaCl_2_, 3.25 KNO_3_, 1.25 MgCl_2_, 0.5 (NH_4_)_2_SO_4_, and 0.2 NH_4_NO_3_, in mM concentrations, and a final pH of 4.5. After three days, the seedlings were transferred for 24 h onto the same medium containing 16 ppm Al^3+^ ions in the form of AlCl_3._ Next, after washing of Al^3+^ ions, seedlings were placed again into the base solution for 48 h to induce root regrowth. The roots of tolerant forms regrow in the opposite to the roots of sensitive forms. The control in this experiment was seedlings grown in medium without Al-ions [37]. The experiment was run in a growth chamber (Pol-Eko Aparatura, ST500 B40 FOT10) at 25 °C with a 12 h day/night photoperiod and a light intensity of 40 W∙m^−^^2^. The seedlings’ aluminum tolerance was assessed on the basis of the regrowth rate of roots stained prior to evaluation in 0.1% Eriochrome cyanine R within 10 min (Figure 1). For antioxidant activity estimation and proteomic analysis, the root tips (0.3–0.4 cm) from 7-day old days seedlings, both exposed and non-exposed to Al^3+^ ions, were excised. The root staining was omitted in this case. The results were based on four independent biological experiments. 

### 4.2. Antioxidant Potential Determination

Antioxidant potential was determined using two radicals, stable anion radical DPPH*^−^ (2,2-diphenyl-1-picrylhydrazyl radical) and cation radical ABTS*^+^ (2,2′-azino-bis (3-ethylbenzo thiazoline-6-sulphonic acid radical) (Sigma-Aldrich Ltd., Poznań, Poland), and was expressed in (μmol/mg) of Trolox equivalents (6-hydroxy-2,5,7,8-tetramethychroman-2-carboxylic acid) (Sigma-Aldrich Ltd., Poznań, Poland) [31]. A UV-2101PC UV-Vis scanning spectrophotometer (Shimadzu, Kioto, Japan) was used for absorbance measurements. The root tips were mashed into powder in liquid nitrogen, extracted in 80% methanol (MetOH) (100 mg/1 mL) at room temperature for 2h, and centrifuged. The reaction mixture consisted of 200 (μL) of the root extract and 3.2 (mL) of DPPH*^−^ in 80% MetOH (10 mg/25 mL). Absorbance was measured at 515 nm after 20 min. The ABTS*^+^ cation radical was prepared by oxidation of 7 mM ABTS water solution by 2.45 mM potassium persulfate overnight (16 h), at room temperature in the dark, and then dilution with 80% methanol to absorbance ca. 0.70 at 734 nm. The reaction mixture consisted of 50 μL of the root extract and 3.7 mL ABTS *^+^, and the measurement was performed at 734 nm after 6 min. 

One-way analysis of variance (ANOVA) was performed with Addinsoft 2020 XLSTAT (New York, NY, USA. https://www.xlstat.com, accessed on 20 December 2021). A Tukey HSD (honestly significant difference) multiple comparison test was used to identify statistically homogeneous subsets at α = 0.05.

### 4.3. Proteomic Studies

#### Phenol-SDS Buffer Extraction with Sonication (PSWS)

The phenol extraction of proteins was carried out as described by Hurkman and Tanaka [76]. Root tissue (300 mg) was ground in a mortar in the presence of liquid nitrogen and transferred to a 1.5 mL Eppendorf tube. Proteins were extracted with 3 mL of SDS buffer (30% sucrose, 2% SDS, 0.1 M Tris-Cl, 5% β-mercaptoethanol, and 1 mM phenylmethylsulfonyl fluoride (PMSF), pH 8.0) by triple sonication for 15 s at 60 amps. After sonication, 0.8 mL of Tris buffered phenol was added to the mixture and vortexed for 10 mins at 4 °C. The set was centrifuged at 14,000× *g* for 5 min at 4 °C, and the phenolic phase was collected and re-extracted with 0.8 mL SDS buffer and shaken for 5 min. Centrifugation was further repeated using the same settings, with the phenolic phase collected and precipitated overnight with four volumes of 0.1 M ammonium acetate in methanol at −20 °C. The precipitate obtained by centrifugation at 14,000× *g* for 10 min at 4 °C was washed thrice with cold 0.1 M ammonium acetate and finally with cold 80% acetone. The pellet was dried and resuspended in 100 μL of sample buffer (Biorad) and used for further analyses. Protein concentrations were quantified using the Bradford protein assay method, using BSA as a standard.

### 4.4. Two-Dimensional Electrophoresis (2-DE)

IPG strips (ReadyStripTMIPG, pH = 3–10, 17cm, Biorad) were passively rehydrated overnight with rehydration sample buffer (7M urea, 2 M thiourea, 4% CHAPS, 0.5% IPG Buffer, 20 mM DTT, 0.002% bromophenol blue) containing 250 μg of isolated protein. First-dimension 

Isoelectric focusing (IEF) was conducted using the following parameters: step 1-gradient volt, 1000 V for 60 mins, step 2-gradient volt, 12,000 V for 60 min, step 3-constant volt, 12,000 V for 25,000 volt hours, and step 4-constant volt, 1000 V for 60 min. All steps were performed at 20 °C using IEF 100 (Hoefer Scientific Instruments, San Francisco, CA, USA). Following IEF, the strips were reduced with 130 mM DTT in 10 mL of equilibration buffer (29.3% glycerol, 75 mM Tris-Cl, 6 M urea, 2% SDS, pH 8.8) for 15 min and alkylated with 135 mM iodoacetamide in 10 mL equilibration buffer for 15 min. The 2-DE was performed according to the Laemmli [77] protocol in lab cast 1.5 mm 12.5% (w/v) polyacrylamide gels using a Hoefer SE 600 Chroma Vertical Electrophoresis System (Hoefer Scientific Instruments, San Francisco, CA, USA). The following program was implemented: 15 mA/gel for 15 min and 30 mA/gel for 90 min in Tris glycine-SDS running buffer. Three gels, one from each independent biological replication, were used for the identification of differential proteins. The gels were stained with 0.1% (w/v) Coomassie brilliant blue R-250 (Sigma-Aldrich Ltd., Poznań, Poland) overnight, destained, and stored in 5% acetic acid at 4 °C for further analysis [78].

### 4.5. Analysis of 2D PAGE Gel Images

Stained gels were digitalized, annotated, and analyzed using Image Master 2D Platinum 7.0 software (GE Healthcare). Data were normalized by expressing abundance as relative volume (% vol). A difference in protein expression was accepted when the Student’s *t*-test was at a significance level of 99% (*p* ≤ 0.01). Spots were only accepted as present or absent if they were present or missing in all four gels from control or treated material/groups. Moreover, in the case of spots appearing only in the control (silenced) or stressed (induced) roots, only those with a signal intensity value >0.2 were considered as significant. The gels obtained for both NT lines were compared visually for identification of the identical spots showing the highest signal intensity changes.

### 4.6. Protein Identification by Mass Spectrometry and Database Search

To identify the protein content in interesting spots, gel pieces were manually cut out and subjected to a standard procedure during which proteins were reduced with DTT, alkylated with iodoacetamide, and digested overnight with trypsin (Sequencing Grade Modified Trypsin, Promega, Madison, WI, USA). The analyses were made by the Mass Spectrometry Laboratory, Institute of Biochemistry and Biophysics Polish Academy of Science (MS Lab IBB-PAN, Warsaw, Poland). The peptide mixtures were analyzed by liquid chromatography coupled to tandem mass spectrometry (LC-MS-MS/MS) with a classic mass spectrometer and LTQ (linear trap quadrupole ion trap-Orbitrap) (Thermo Electron Corporation, San Jose, CA, USA). Briefly, the peptide mixture was applied to an RP-18 precolumn (nanoACQUITY Symmetry^®^ C18, Waters, Milford, CT, USA) using water containing 0.1% (*m/v*) formic acid (FA) as a mobile phase and then transferred to a nano-HPLC RP-18 column (nanoACQUITY BEH C18, Waters) using an acetonitrile gradient (0–60%, *v/v*, in 120 min) in the presence of 0.05% (*m/v*) formic acid with a flow rate of 0.25 mm^3^ min^−^^1^. The column outlet was directly coupled with the ion source of the spectrometer working in the regime of data dependent MS to MS/MS switch.

After pre-processing the raw data with the Mascot Distiller v. 2.6.1.0 software (Matrix Science, London, UK), the obtained peak lists were used to search the non-redundant protein database of the National Centre for Biotechnology Information (NCBI) using the Mascot search engine (v. 2.5.1, Matrix Science). The taxonomic category selected was *Triticum aestivum*. Only peptides passing a Mascot-defined expectation value of 0.05 were considered as positive identifications [78]. The functional networks of differentially expressed proteins were constructed using the STRING database [32]. 

## Figures and Tables

**Table 1:** Root regrowth (cm) and antioxidant potential of triticale root tips from control seedlings and from seedlings 48 h after 16 (ppm) Al treatment. The total antioxidant capacity of tolerant (L198) and sensitive (L444 and L17) genotypes was expressed as the µmol Trolox equivalent antioxidant capacity (TEAC) per mg of root tip tissue.

Line	Control/Stress	Root Regrowth (cm)	DPPH*^−^ (μmol TEAC/mg)	ABTS*^+^ (μmol TEAC/mg)
L198	control	-	15.264 ± 1.17 a	8.706 ± 0.39 ab
L198	stress (16ppm Al)	0.3–2.5	15.786 ± 1.29 a	9.170 ± 0.39 a
L444	control	-	13.854 ± 1.00 a	8.072 ± 0.11 b
L444	stress (16ppm Al)	no regrowth	16.643 ± 3.34 a	9.124 ± 0.25 a
L17	control	-	12.544 ± 2.05 a	8.086 ± 0.58 b
L17	stress (16ppm Al)	no regrowth	12.602 ± 0.9 a	8.870 ± 0.10 ab

a,b—statistically different mean values (*p* < 0.05); DPPH*^−^—(2,2-diphenyl-1-picrylhydrazyl); ABTS*^+^—(2,2′-azinobis-(3-ethylbenzothiazoline-6-sulfonic acid).

**Table 2:** The number of identified protein spots on 2-DE gels from analyzed root tip proteomes of tolerant (L198) and susceptible (L17 and L44) triticale lines, in accordance with the t-test with probability *p* ≤ 0.01. In parenthesis are the number of spots selected with double criterion: high probability (*p* ≤ 0.01) and at least two-fold difference in spot intensity (≥2×). For induced and silenced protein spots, the second criterion was spot relative intensity ≥ 0.2.

Line	Tolerant	Sensitive		L17-L444
Spots Characteristic	L198	L17	L444	Common Spots (with *p* ≤ 0.01)
Total number	579	584	602	
Upregulated	0	23 (14 ≥ 2×)	23 (18 ≥ 2×)	13
Downregulated	0	21 (8 ≥ 2×)	8 (0 ≥ 2×)	2
Silenced upon Al^3+^	0	12 (3 ≥ 0.2)	9 (1 ≥ 0.2)	3
Induced upon Al^3+^	0	15 (9 ≥ 0.2)	3 (0 ≥ 0.2)	2

**Table 3:** Identification of differential protein spots from 2-DE gels obtained by separation of seedling root tip proteins extracted from Al-sensitive triticale lines, L17 and L444, after stress release. Protein spots were chosen according to the double criterion of *p* ≤ 0.01 and difference in spot intensity ≥ 2, and for induced or silenced proteins the criterion of relative spot intensity ≥ 0.2 was decided. Protein spots were detected using Image Master 2D Platinum 7.0 software, followed by MS-MS separation and further identification, characterization, and quantitation using Mascot Distiller v. 2.3 software.

Spot No.	Pathway/Protein Name	UniProt ID	Mascot Score	Mass	pI	^1^ MP	Fold Changed	
							L17	L444
	Cell signaling							
1	Calmodulin	P04464	25	16,893	4.9	1	+2.52	* n.s.
	Metabolic pathway							
2	ATP synthase subunit alpha, mitochondrial	P12862	201/96	55,515	6.6	4	+3.54	+2.73
3	Adenosylhomocysteinase	P32112	34	54,086	7.85	1	−2.00	−0.83
4	Phosphoglycerate kinase, cytosolic	P12783	372	42,153	5.6	7	+3.13	+5.00
5	Fructose-1,6-bisphosphatase	P09195	24	44,703	7.1	1	+2.03	* n.s.
	Metabolic pathway/Flavonoid metabolism							
6	Flavone O-methyltransferase 1	Q84N28	1053	39,177	5.7	22	+7.61	see Table 3
	Methyl cycle							
7	S-adenosylmethionine synthase	B0LXM0	448	43,609	5.51	7	+2.91	n.s.
	Protease inhibitor							
8	Serpin-Z1C	Q9ST58	185	42,969	5.45	4	+2.03	+1.72
	Protein degradation/cell signaling							
9	Ubiquitin	P69326	42	8648	7.2	1	+2.01	+3.6
10	Ubiquitin	P69326	38	8648	6.79	1	+2.05	n.s.
11	Ubiquitin	P69326	55	8648	7.25	1	+2.43	n.s.
	Protein synthesis							
12	Protein disulfide-isomerase	P52589	113	56,726	4.9	4	+2.51	+3.61
13	Eukaryotic initiation factor 4A	P41378	114	47,183	5.25	2	+4.12	+1.92
14	Protein disulfide-isomerase	P52589	81	56,726	5.11	3	+2.65	+1.78
	Stress related							
15	Dehydrin COR410	P46524	50	28,166	6.9	1	+2.50	+1.34
16	Oxalate oxidase	P26759	341/394	23,711	6.35	5	+2.75	+5.00
17	Glutathione S-transferase	O04437	218/474	24,022	6,2	11	+2.85	+4.52
18	1-Cys peroxiredoxin	Q6W8Q2	298	24178	6	5	+2.87	+3.51
	Transcription control							
19	Splicing factor U2af large subunit B	Q2QKB4	334	60,720	5.2	4	+2.01	+1.51
20	DNA-directed RNA polymerase subunit beta	Q9XPS9	14	170,794	6.25	1	+2.05	n.s.
	Transport							
21	Mitochondrial outer membrane porin	P46274	16	28,944	6.5	1	+2.33	+1.62
	Lignin synthesis							
22	^2^ DIMBOA1b, chloroplastic	Q1XH05	56	64,898	5.25	2	n.s.	+2.87
23	^2^ DIMBOA 1c, chloroplastic	Q1XH04	80	64,980	5.4	2	n.s.	+2.16
	Unassigned peptides							
24	Unassigned peptide	-	-	-	-	-	−5.2	n.s.
25	Unassigned peptide	-	-	-	-	-	+2.65	n.s.

^1^ MP—matched peptides; ^2^ DIMBOA—4-hydroxy-7-methoxy-3,4-dihydro-2H-1,4-benzoxazin-2yl beta glucosidase; * n.s.—protein spot not significantly changed.

**Table 4:** Aluminum ion-responsive proteins from seedling roots of Al-sensitive triticale lines, L17 and L444, present in roots of control (proteins silenced upon Al^3+^) or in roots after the stress removal (proteins induced upon Al^3+^). Double cut-off criterion *p* ≤ 0.01 and relative spot intensity ≥ 0.2 were used. The relative intensity of protein spots on the gels are shown.

Spot No.	Pathway/Protein Name	UniProt/String (mloc) ID	Mascot Score	Mass	pI	^1^ MP	^2^ Spot Intensity	
							L17	L444
	Cell division/Cytoskeleton							
26	Tubulin beta-3 chain	Q9ZRB0	791	50,555	4.9	18	−0.21	* n.s.
27	Tubulin alpha chain	Q9ZRB7	2061	50,396	4.95	26	−0.20	n.s.
	Lignin synthesis							
28	^3^ DIMBOA 1b, chloroplastic	Q1XH05	328	64,898	5.55	7	+0.21	n.s.
29	^3^ DIMBOA 1b, chloroplastic	Q1XH05	215	64,898	5.45	4	+0.23	n.s.
	Metabolic pathway							
30	Phosphomannomutase	Q1W374	505	28,405	6	11	n.s.	−0.20
	Metabolic pathway/Flavonoid metabolism							
31	Flavone O-methyltransferase 1	Q84N28	1053	39,177	5.7	22	see Table 2	+0.26
	Methyl cycle							
32	Adenosylhomocysteinase	P32112	137	54,086	6.8	3	−0.20	n.s.
	Protease inhibitor							
33	Ubiquitin	P69326	67	8648	7.6	1	+0.21	n.s.
34	Ubiquitin	P69326	70	8648	6.45	1	+0.22	n.s.
35	Ubiquitin	P69326	40	8648	7.25	1	+0.28	+0.26
	Unassigned peptides							
36	Unassigned peptide	-	-	-	-		n.s.	−0.24

^1^ MP—matched peptides; ^2^ relative spot intensity on the gel; (−) silenced; (+) induced; ^3^ DIMBOA—4-hydroxy-7-methoxy-3,4-dihydro-2H-1,4-benzoxazin-2yl beta glucosidase; * n.s.—protein spot visible on the gel but not changed in a significant manner.

## Data Availability

Data available upon request.

## Supplementary Materials

The following supporting information can be downloaded at: https://www.mdpi.com/article/10.3390/plants11020165/s1, Figure S1 The stages of the experiment: (A) triticale seeds germinating on the polyethylene grid tray; (B) 5th days old triticale seedlings; (C) the growth chamber view; Figure S2 Protein separation by 2-DE on gels stained in Coomassie Brilliant Blue (A-F). Proteome of L17 Al-sensitive line control (A) and 48h after Al stress treated (B); proteome of L444 Al-sensitive line control (C) and 48h after Al stress treated (D); proteome of L198 Al-tolerant line control (E) and 48h after Al stress treated (F). The differential protein spots, which were common for both studied Al-sensitive lines, are marked in red on gel pictures of L17 and L444 line (control vs. Al-treated). The differential protein spots, which were characteristic only for one studied Al-sensitive lines, are marked in green on gel pictures of L17 and L444 line (control vs. Al-treated). The Image Master 2D Platinum 7.0 software was used for differential spots identification; Figure S3 Comparison in: (A) number of up/down-regulated and silenced/induced proteins and (B) number of common protein spots according to established criterions (*p* ≤ 0.01 and difference in spot intensity ≥ 2-fold or 0.2 relative intensity of silenced/induced proteins).
