# Supplementary material for: Aluminum Stress Induces Irreversible Proteomic Changes in the Roots of the Sensitive but Not the Tolerant Genotype of Triticale Seedlings
Source: Plants (Basel). 2022 Jan 8;11(2):165. doi: 10.3390/plants11020165 (PMC8781804; doi:10.3390/plants11020165)
Supplement: Supplementary file 1 [file plants-11-00165-s001.zip › plants-1492761-supplementary.pdf]

Supplementary Materials

Aluminum Stress Induces Irreversible Proteomic Changes in the Roots of the Sensitive but Not the Tolerant Genotype of Triticale Seedlings

Agnieszka Niedziela [1]*, Lucyna Domżalska [2], Wioletta M. Dynkowska [1] and Markéta Pernisová [3,4], Krystyna Rybka [1]*

[1] Department of Biochemistry and Biotechnology, Plant Breeding and Acclimatization Institute-National Research Institute in Radzików, 05-870 Blonie , Poland; w.dynkowska@ihar.edu.pl
[2] Polish Academy of Sciences Botanical Garden – Center for Biological Diversity Conservation in Powsin, Prawdziwka 2, 02-973, Warsaw, Poland; lucyna.domzalska@gmail.com
[3] Plant Sciences Core Facility, Mendel Centre for Plant Genomics and Proteomics, Central European Institute of Technology (CEITEC), Masaryk University, Kamenice 5, 62500 Brno, Czechia; marketa.pernisova@ceitec.muni.cz
[4] Laboratory of Functional Genomics and Proteomics, National Centre for Biomolecular Research, Faculty of Science, Masaryk University, Kotlářská 2, 61137 Brno, Czechia.
* Correspondence: a.niedziela@ihar.edu.pl (A.N.); k.rybka@ihar.edu.pl (K.R.);
Tel.: +48-227-334-535 (A.N.); +48-227-334-537 (K.R.)

Content:

Figure S1 The stages of the experiment: (A) triticale seeds germinating on the polyethylene grid tray; (B) 5th days old triticale seedlings; (C) the growth chamber view.

Figure S2 Protein separation by 2-DE on gels stained in Coomassie Brilliant Blue (A-F). Proteome of L17 Al-sensitive line control (A) and 48h after Al stress treated (B); proteome of L444 Al-sensitive line control (C) and 48h after Al stress treated (D); proteome of L198 Al-tolerant line control (E) and 48h after Al stress treated (F). The differential protein spots, which were common for both studied Al-sensitive lines, are marked in red on gel pictures of L17 and L444 line (control vs. Al-treated). The differential protein spots, which were characteristic only for one studied Al-sensitive lines, are marked in green on gel pictures of L17 and L444 line (control vs. Al-treated). The Image Master 2D Platinum 7.0 software was used for differential spots identification.

Figure S3 Comparison in: A) number of up/down-regulated and silenced/induced proteins and B) number of common protein spots according to established criterions ($p \leq 0.01$ and difference in spot intensity ≥ 2-fold or 0.2 relative intensity of silenced/induced proteins).
When the criterion of probability has been weakened from 99% to 95% and twofold difference was not considered, more than 80% of differential proteins were common between control and stress-treated roots tips proteomes of Al-sensitive seedlings.

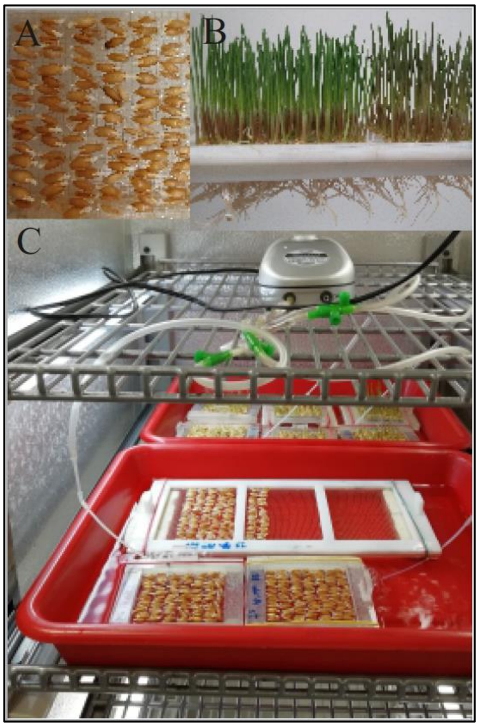

Figure S1. The stages of the experiment:
(A) triticale seeds germinating on the polyethylene grid tray;
(B) 5th days old triticale seedlings;
(C) the growth chamber view.

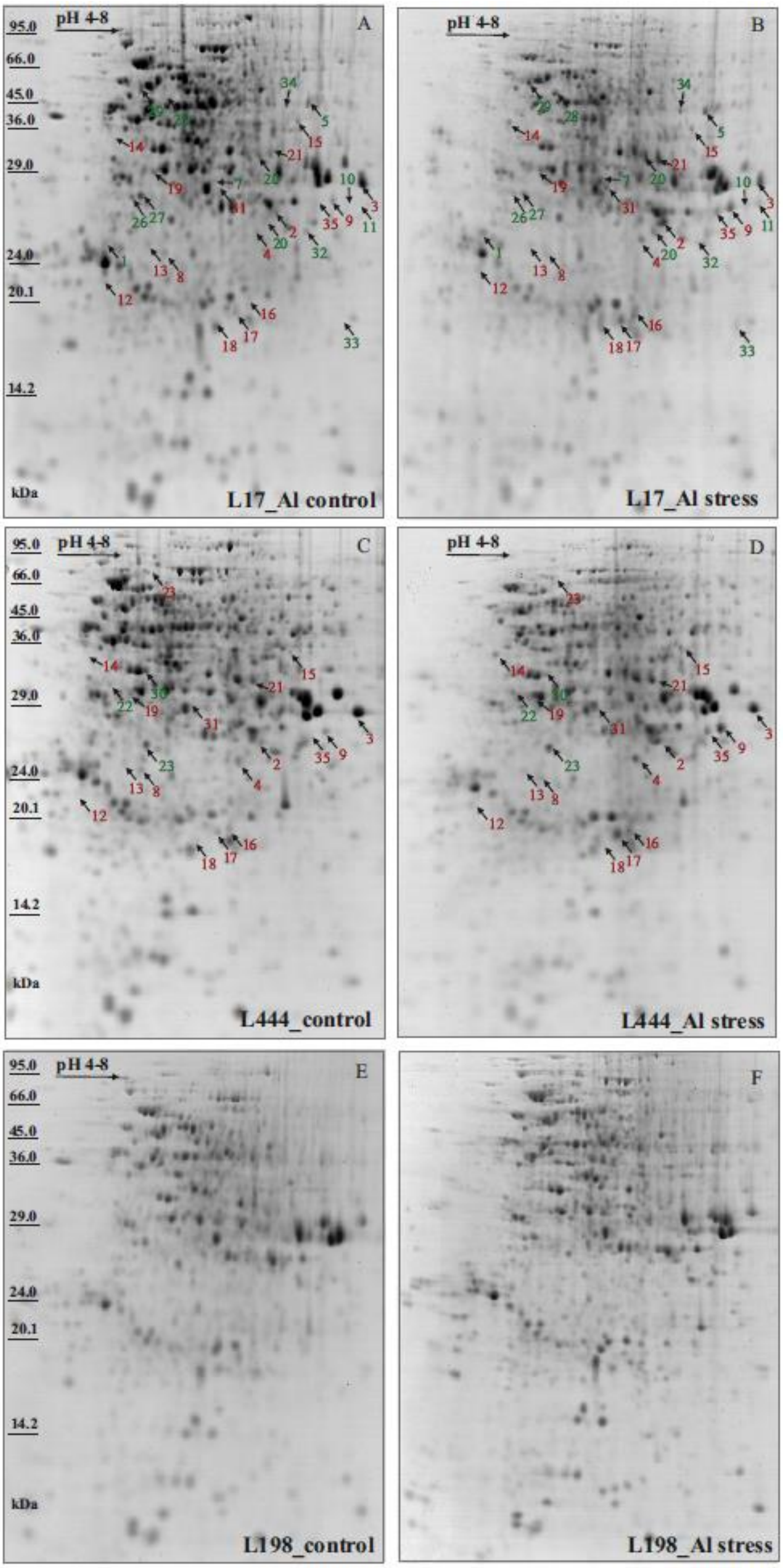

Figure S2. Protein separation by 2-DE on gels stained in Coomassie Brilliant Blue (A-F). Proteome of L17 Al-sensitive line control (A) and 48h after Al stress treated (B); proteome of L444 Al-sensitive line control (C) and 48h after Al stress treated (D); proteome of L198 Al-tolerant line control (E) and 48h after Al stress treated (F). The differential protein spots, which were common for both studied Al-sensitive lines, are marked in red on gel pictures of L17 and L444 line (control vs. Al-treated). The differential protein spots, which were characteristic only for one studied Al-sensitive lines, are marked in green on gel pictures of L17 and L444 line (control vs. Al-treated). The Image Master 2D Platinum 7.0 software was used for differential spots identification.

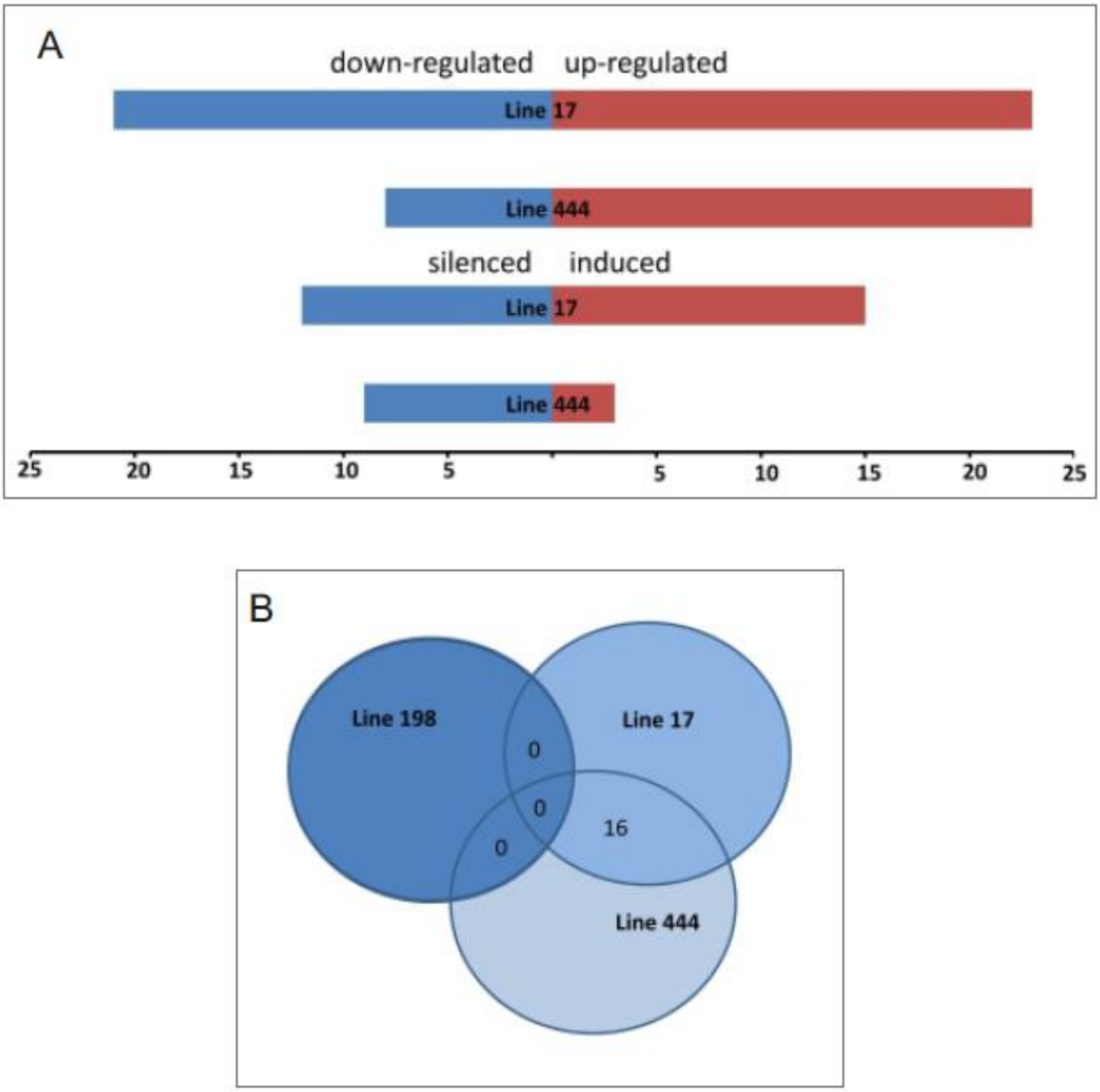

Figure S3. Comparison in: (A) number of up/down-regulated and silenced/induced proteins and (B) number of common protein spots according to established criterions ($p \leq 0.01$ and difference in spot intensity ≥ 2-fold or 0.2 relative intensity of silenced/induced proteins). When the criterion of probability has been weakened from 99% to 95% and twofold difference was not considered, more than 80% of differential proteins were common between control and stress-treated roots tips proteomes of Al-sensitive seedlings.
